# To the Editors of the London Medical and Physical Journal

**Published:** 1816-07

**Authors:** John Hoare

**Affiliations:** Warminster


*To the Editors of the London Medical and Physical Journal.*

GENTLEMEN,

IF you think the subsequent statement of vaccination worthy of a place in your Journal, I shall be happy to see it inserted; not that I suspect your readers, in general, have any doubt of its entire success, but because it bears such a contrast to that of Mr. Walker's, which appeared in your Number for February. I am, Gentlemen,

*Warminster;* Your obedient humble Servant,

*May* 22, 1816. JOHN HOARE.

---

Since October last, I have been more than commonly engaged in vaccination, and principally amongst the poor, for whom, in order to extend it as much as possible, I gave information it should be done gratis. Their prejudices being great as yet, I did not expect much success; however, of this class alone I vaccinated nearly 160, when, about three months ago, the small-pox made its appearance. Vaccination (chiefly amongst the poor) experienced a shock, as it was natural to expect: myself, with the rest of the medical men here, were determined not to inoculate, and used our utmost efforts (as well as the parish officers) to prevent its being brought into the town, but in vain;—the women themselves propagated the disease with stocking needles, and soon spread it over the place. By this time, many of those women whose children had been vaccinated, began to be afraid, and requested I would *inoculate* them, which I did with pleasure, to about thirty, of various ages, and as various in the times of their vaccination, for some of them were done six, seven, eight, and nine years ago; and, I am happy to say, *not one* experienced the slightest affection in the system. The inoculated part certainly had the appearance of rising pustules, but no formation of matter. I offered to do it to as many as chose, but the rest would not try the experiment, being well satisfied; and the prejudices, which have been strong, and were great even prior to this period of vaccination, are immensely subsided.

N.B.—Notwithstanding the serenity of this season, out of about 150 which were inoculated for small-pox, five died!

---

*For the London Medical and Physical Journal.*

